# The Relation of Tuberculosis to Municipal and Industrial Life1Read at the annual session of the Board of Trade, Amsterdam, N. Y., Monday evening, January 14, 1907.

**Published:** 1907-05

**Authors:** Charles Stover

**Affiliations:** Amsterdam, N. Y.


### BUFFALO MEDICAL JOURNAL.

VOL. LXII. MAY, 1907. No. 10

### ORIGINAL COMMUNICATIONS.

### The Relation of Tuberculosis to Municipal and Industrial Life.[1]

By CHARLES STOVER, M. D., Amsterdam, N. Y.

WHEN it is remembered that there are annually 150,000 deaths from tuberculosis in the United States, that there are in the state of New York at this moment not less than 35,000 living victims of this disease, some consideration, first of the appalling waste of human life, and second of the means for its prevention, cannot fail to interest us.

Ten per cent. of the deaths in the United States from all causes are from tuberculosis. The decade from 1896 to 1907, out of an annual average of 126,667 deaths in New York state, 13,365 were from this disease about 10½ per cent. In the city of Amsterdam for the same period there was an average of 392.1 deaths, of which 31.3 were tubercular—about 10 per cent. For the same period in Montgomery county as a whole, there were on the average 50.9 deaths annually, which indicates that about three-fifths of all the cases occur in the city of Amsterdam, although the population is but one-half or less than that of the county. The loss to the state is the greater because these lives are given up, not at the end, but rather at the beginning, or in the midst of their active careers. Thus Osler states that the ancient opinion of Hippocrates is correct—namely, that the greatest number of cases occur between the ages of 18 to 35 years, although no age is exempt, from the suckling to the octogenarian.

GOVERNMENT ISOLATION HOSPITALS.

Recognising the communicability of this disease, state and national governments have undertaken the cure of tuberculosis in isolated hospitals. New York state has Raybrook, in the Adirondacks, with accommodations at present for about one

1. Read at the annual session of the Board of Trade, Amsterdam, N. Y., Monday evening, January 14, 1907.

hundred and fifty cases. The policy of the state is to provide only for incipient consumption, or to put it in another way, for those most easily curable, so that the greatest number possible at the least expense, may be returned to continue their vocations. It is to relieve the state of pensioners, not to provide an asylum for the incurable. Unfortunately very many cases cannot avail themselves of a change of climate or a health resort or a sanitarium or tent colony life. It is stated as a fact that 25 per cent. of the families in the United States are living on $400 a year or less. Now, from out of these homes and among these families quite a large percentage of tubercular patients come, hence they must remain under the care and observation of their family physician until relieved by art or released by death. States should take cognisance of this fact, for a nation's humanity and wisdom is nowhere better shown than in the care and safety it gives its helpless and dependent people. While the state is thus doing something in the care of victims of tuberculosis, can it be said that municipalities and corporations are doing all that can be done in this direction? In order to answer this inquiry we will later refer to some of the causes that are recognised as contributing to the production of tuberculosis.

### PURE AIR OF GREATEST IMPORTANCE.

It is unfortunate that a popular belief in the incurability of tuberculosis so widely exists. There has been an unmistakable decrease in the disease during the past decade. The records of New York city indicate this. Massachusetts, that was a hotbed of tuberculosis for so many years, has reduced the death rate very considerably in this particular. Glasgow, that keeps very careful statistics, has shown a very extraordinary fall in the death rate from tuberculosis. There are scores of people among us who have recovered by change of climate, change of occupation, or change of environment. There are scores of people among us who are destined to die of tuberculosis if changes in these respects are not made for them. If the city cannot go to the country, cannot something of the country be brought to the city to help these unfortunates? Pure air is a vital necessity, but of all needs, most sacrificed in the evolution of our civilization. Everybody knows that in badly ventilated rooms the air acquires a bad odor, especially noticeable on entering, and that persons upon remaining, sooner or later suffer.

According to Professor James, of Columbia university, New York, the principal symptoms are a feeling of heaviness and oppression, headache, drowsiness, malaise, vertigo, tinnitus aurium (ringing in the ears), nausea and faintness. If the conditions are maintained for a longer time, there is a loss of appetite, coated tongue, indigestion, nausea, constipation, and nervousness, and subsequently, if the evil be persisted in, the general health may be seriously impaired, secondary anemia and malnutrition being pronounced features, together with a general increased susceptibility to the infectious diseases. Consider for a moment that an individual breathes from 14 to 20 times per minute, that is, he gets about 1,000 feeds of air in an hour. The primary object is to get oxygen to vitalise the system. Reflect that in rooms not equipped with proper ventilation, the occupants breathe the expired air over and over, not of their own bodies alone, but that of others. To this foul air add the impurities, the gases, the dust and dirt of industrial life, and is it any wonder that pulmonary disease is frequent? But the evil does not end there: the lowered vitality from imperfect oxygenation of the blood leads to many other ills in other tissues and other organs. The public school presents the same conditions operating upon very susceptible subjects.

### SCHOOL VENTILATION MUST BE ABSOLUTE.

Some English doctor shows that where mechanical ventilation was applied to school rooms, the carbonic gas was three-fifths, the organic matter was one-seventh, and the micro-organisms less than one-ninth of what was found in schools ventilated by the usual methods. It may be asked what is considered good ventilation in a school room. Dr. D. F. Lincoln, a recognised authority on this subject, says the air should be furnished in a fresh volume of from 40 to 100 cubic meters hourly to each scholar, that is, 1,400 to 3,500 cubic feet. Professor Howell of Johns Hopkins, also places the necessary amount per hour at 100 cubic meters. That is to say, if an individual occupied a room 20x15 and 10 feet high, there would be in it sufficient air for one hour, if it were possible to utilise it all.

If the room is spacious so that each pupil has 300 cubic feet of space the whole air-contents of the room might be evacuated from five to twelve times an hour. If the room, however, is small, say 200 cubic feet per head, the change must go on faster—the entire contents must be changed every 3½ minutes. Now this is done without causing a draught. But when the room is crowded, in order to keep up the standard, suppose the air is changed once in four minutes, there will then be a very great draught. Therefore, a closely packed room cannot be well ventilated because the inmates cannot stand the draught. The conclusion then is that each pupil should have about fifteen square feet of floor space, or better still, twenty.

One of this committee had his attention drawn to the fact that many children in the East Main street school, of this city, had sore throats, due to exposure in the winter time by open windows raised for ventilation. Investigation showed that the janitor had been instructed by a member of the board of education to close the ventilators because too much coal was consumed.

There is occasion for a better system for cleaning the floors of the public school buildings. One of the janitors says that the occupation of the room limits opportunity during the daytime, while the absence of illumination makes it impossible at night. Moreover the dry sweeping fails to remove the dirt satisfactorily. Moist sawdust might present some advantages. Father Browne, with his accustomed enterprise, has had installed at St. Mary's church and institute, a dustless pneumatic apparatus similar to that frequently seen in the large New York hotels.

### CROWDED TENEMENTS A GREAT EVIL.

The pinching poverty that obliges so many thousands to live in crowded tenements is the cause of deprivation of air. Thus, in Glasgow it was shown that in acute diseases of the lungs the death rate was as high as 9.85 in the smallest tenements, and as low as 3.28 in the largest. The rate is 3 to 1. In the British army in a period of 1830-1846, the mortality from tuberculosis was 7.86 per 1,000; in 1859-1866, when an increased cubic space per head was made, it fell from 7.86 to 3.1 per thousand.

Dr. T. Mitchell Prudden, New York, says infectious diseases of the respiratory organs are steadily increasing; as people are more and more huddled together in offices, dwellings, conveyances, and places of public assemblage, a large part of these diseases are directly traceable to infectious cast-off material in the air.

In some of the broom factories in this city the moistening of the dusty broom corn lessened the baneful effect of dust upon the respiratory tract. Likewise the substitution of wet for dry grinding was of great benefit to the grinders employed in the spring shops of the city. Two years ago in one of the industries of our city there occurred an unusual number of pulmonary hemorrhages. It was thought that a very dry dust resulting from the manufacturing process carried on, was the cause of the trouble. The proprietors gave one of our physicians a free hand to suggest and apply a remedy. Unfortunately there was no precedent to be precisely applied, but by experience and liberal expenditure of money, the introduction of suction and blowing apparatus has steadily improved the sanitary conditions, and pulmonary hemorrhages are no longer conspicuous. There can be no doubt about the life saving effects of these industrial changes.

It is summed up this way: the greater number of our people must live indoors and be more or less crowded together; conditions of health demand pure, fresh, unbreathed air; the air of assembly rooms is insufficient, more should be introduced, while foul air is removed.

COMMERCE AND INDUSTRIES NEED REGULATING

Right here we are confronted by another difficulty—namely, the contamination of the air by commerce and municipal enterprise, another penalty paid for being civilised. Every municipality should be encouraged in an honest effort to maintain clean streets. Dry sweeping, as has been recommended for asphalt pavements, probably does prolong the life of the pavement, but it only displaces the dust and creates a nuisance to annoy pedestrians and adjacent house dwellers. Watering, of course, effectually settles the dust, but on stone surfaced roads it must be regularly repeated and this results in a muddy street, which is another nuisance. Moreover, it involves a considerable expense that few municipalities will be willing to incur, for it is assumed that individual effort cannot be relied upon. One man sprinkles his street front, but suffers from the dust of his less energetic neighbor.

Some authorities advocate the use of crude petroleum, or thin tar applied on the surface or incorporated in the upper layers of the macadam road. Some of you have seen this in your auto trips about Saratoga and Albany. In California, where petroleum is cheap, it is said to be very satisfactory. In respect to sprinkling the brick pavement, that seems to be so generally popular, there is a conspicuous advantage. It can be sprinkled and even kept continuously moist without that disintegration of substance that marks asphalt when watered. It is submitted that no pavement can be freed from its dust by dry cleaning alone; sprinkling must be supplemental.

THE ROLE OF BACTERIA AND DUST.

Professor Tyndall, and Monsieur Jean Binot, both carried on experiments to determine the bacterial limit in the air, ascending Mt. Blanc for the purpose of observation. Near the mountain top it was found that no or few bacteria were found. The air of the country has fewer than that of cities; the open parks in cities are better than the slums near by. The ordinary streets are much like the slums; some of us can shun the latter, but we cannot shun the street. On a blistering summer day or in blustery March, a thimbleful of the pavement dust may contain from 900 to 160 millions of bacteria. This enumeration fails to indicate the significance of this statement, it marks the quantity but not the quality. Some of these bacteria are very potent for mischief. Think for a moment how many consumptives expectorate in the streets! The bacillus of tuberculosis has been found alive in sputa when kept dry for ten months. A rigid enforcement of the law against spitting in public places ought to be applied.

The whirlwinds of dust, formerly occasional, now made perpetual by the modern automobile and trolley car, permeate the dwellings so that few houses are freed from constant ravages by dust. It has been observed that the upper stories of houses and public buildings are the freest from this nuisance, a sanitary argument for the skyscraper.

When Morrell Mackenzie toured the United States he came to the conclusion that the dust of the cities, due to imperfectly cleaned streets, was the chief cause of the widely prevalent catarrhs that marked a national disease.

As another means of lessening tuberculosis the close inspection of the milk supply by our sanitary officials is to be encouraged. The relations of human and bovine tuberculosis cannot be ignored. There has been some question as to the communicability of the one by the other, the balance of authority being in the affirmative. But apart from this, inasmuch as pure milk is essential to infant feeding, it is necessary that thousands of infants having a predisposition to tuberculosis, shall be safeguarded by good nourishment during the early years of life, for be it remembered that something besides the germ of the disease is essential to develop tuberculosis, that is, a proper soil. Dr. Austin Flint likened it to a pair of scissors, one blade representing the bacterium, the other blade representing the soil. They must be united to be efficient. Herein lies the whole scheme for the prevention of tuberculosis or any infectious disease. On the one hand, the problem is to exterminate the bacteria, and on the other to raise the vital resistance of the individual by every means that contributes to better health, by pure food, pure air, pure water, pure soil.

### MILK MUST BE PURE.

A Massachusetts agricultural society reported a case where 33 per cent. of the calves fed from the milk of tuberculosis cows succumbed to the same disease. Frequently milk and cheese made from such cows have been shown to be infected with tubercular bacilla. Physicians have traced epidemics of typhoid and scarlet fevers to infected milk. As experience increases, more and more the means of conveying the various infectious diseases are found to be identical, varying only in degree.

Health boards have undertaken to standardise public milk, gauging it by the number of bacteria found in a cubic centimeter.

It should be remembered that some bacteria are always to be found in water and milk, and that not all are harmful. As was said heretofore, the quality of the infection is to be considered. If a standard of 50,000 to the cc. is established no city of the first class would have its supply.

It is too bad to have to state it, but milk with 100,000 per cc. must be allowed to meet the demand. Some milk shows one or two millions to the cc. There is a certified milk (one with an official seal on the container) produced near Canajoharie, marketed in Schenectady and Albany, that in mid-summer showed a test of only 600 to the cc. Now what makes the difference? In a word, cleanliness. It comes from a model dairy, large and airy in construction, with asphalted floors, graded drains, water supply for flushing, groomed cattle washed down on flanks and udders, and tails tied up before milking, attendants' hands washed, white uniforms donned, all containers sterilised by steam, milk put by the milker in strainer in upper floor of milk house (a separate building) and delivered to bottler on lower floor in an aseptic room, where it is sealed and then kept at even temperature.

An epidemic of bowel trouble occurred in a child's hospital in a neighboring city, using certified milk, and the milk was found to have suddenly gone wrong. Investigation showed that while dry hay was being put in the loft of the cow stable, the dust of the hay had caused the outbreak.

### "THE AIR WE BREATHE"

Another source of contamination in the air is found in the burning of soft coal. We get in some measure accustomed to this while in the city, but observe it more particularly when approaching the city from the country, or when the laundry is hung on the line outdoors to dry. If we are enveloped by it for a brief moment by a passing locomotive, or some neighboring smokestacks, we protest, but must perforce continue to breathe it. What is it that we breathe? According to Dr. DeWitt C. Greene, whose interesting paper on this subject in the Buffalo Medical Journal, January, 1907, is liberally drawn upon, the gases that are included in the soot are carbonic oxide, carbonated hydrogen and sulphurated hydrogen. One ton of coal contains about sixteen pounds of sulphur which escapes into the air as sulphurous acid; a portion of this combines with the oxygen of the air to form sulphuric acid.

This is destructive to vegetable and animal tissue, and therefore detrimental to the pulmonary tract of individuals, to the foliage in the landscape, and exceedingly ugly in its effect upon the monuments and buildings of a city. This smoke problem is by no means a new one. "About six hundred years ago when London had a population of only 50,000 the citizens petitioned King Edward I to prohibit the use of soft coal, which is the kind that today makes the smoke for our city, and he responded by making the use of soft coal an offense punishable by death."

At the present time in England, firemen and engineers are liable to a fine if their engines smoke unnecessarily. In Boston there is a law that no boiler will be allowed unless the owner agrees to use hard coal, or a smoke preventer. In Cleveland where so much trouble occurred from smoke of railway locomotives, a record was kept of five different roads running into the city. A chart was used ranging from 0, no smoke, to 100, very dense smoke, and giving the length of time of observation. By judicious firing the amount was reduced from 20 per cent. to 11 per cent. It goes to show what care in stoking will do. New York city has obliged the New York Central & Hudson River Railroad to use anthracite, and very little soft coal was used there before the great coal strike.

Buffalo has an ordinance against smoke from engines but it is not enforced. In England a member of Parliament has proposed to electrify London by producing the current in the midland coal fields and transmitting it to the city. It is estimated that the annual damage by the smoke nuisance in London is $10,000,000. This plan Mr. Lupton thinks would do away with the furnaces and smoke producers and make London the sunniest and most beautiful city in the world. Dr. Greene states that in his neighborhood there is a manufacturing plant which has been in existence for years, during which time no annoyance was experienced by the neighbors on account of smoke. The plant changed hands May 1, 1906, the new firm continuing to use the same bituminous coal, boiler and engine. Soon the amount of smoke emitted became so great at times that the neighbors were compelled to close their windows and send out their laundry. This condition was relieved only by applying to the authorities. It is related by a carpenter who some time ago was employed in Pittsburg in the construction of a hotel, that it was so smoky that each carpenter was given six candles a day that he might be provided with sufficient light.

Anthracite coal, since it is found only in a limited area, principally in Pennsylvania, will always be dearer than bituminous which is scattered throughout many states. It, therefore, is destined to be used. But is it therefore necessary to create a nuisance? Is there necessarily an antagonism here between commerce and sanitation? Munich has the reputation of being the most free from smoke of any manufacturing city in the world. Smoke consumers are used to take up the smoke. In this city are large foundries for making bells, bronzes, iron, machinery, steel wares, and fifty breweries, all using coal. In 1905 Munich had a population of 500,000. It may be that "smoke means industry," but cannot we have industry without smoke?

CONCLUSIONS

Finally, we may recognise the following as our chief weapons in fighting this disease: first, education of the public, particularly of the poorer classes who do not fully appreciate the chief danger of the disease. Second, the compulsory notification and registration of all cases of tuberculosis. The importance of this relates chiefly to the poor and improvident, from whom after all comes the greater danger, and who should be under constant surveillance in order that these dangers may be reduced to a minimum. Third, the foundation in suitable localities by the city and the state, of sanatoria for the treatment of early cases of the disease. Fourth, provision for the chronic, incurable cases in special hospitals. (Osler). These in the main, relate to public hygiene, to official administration. What we have discussed tonight, however, belongs to a higher plane of action—namely, the prevention of disease. In the majority of all cases of tuberculosis the infection is by what is breathed with the air. Can we do anything to make the air better for ourselves and those more or less dependent upon us?

31 Division Street.

